# Takotsubo Cardiomyopathy Following Traumatic Hand Amputation: A Case Report

**DOI:** 10.5811/cpcem.2022.2.55463

**Published:** 2022-07-27

**Authors:** Bastien H. Bacro-Duverger, Ashley Q. Thorburn, Brad D. Denney, John P. Gullett, Maxwell A. Thompson, David C. Pigott

**Affiliations:** * The University of Alabama at Birmingham, Department of Emergency Medicine, Birmingham, Alabama; † The University of Alabama at Birmingham, Division of Plastic Surgery, Department of Surgery, Birmingham, Alabama

**Keywords:** stress cardiomyopathy, takotsubo cardiomyopathy, traumatic amputation, ultrasonography, case report

## Abstract

**Introduction:**

Takotsubo or stress cardiomyopathy is a syndrome of transient left ventricular systolic dysfunction seen in the absence of obstructive coronary artery disease.

**Case Report:**

We describe a case of stress cardiomyopathy diagnosed in the emergency department (ED) using point-of-care ultrasound associated with traumatic hand amputation. The patient suffered a near-complete amputation of the right hand while using a circular saw, subsequently complicated by brief cardiac arrest with rapid return of spontaneous circulation. Point-of-care ultrasonography in the ED revealed the classic findings of takotsubo cardiomyopathy, including apical ballooning of the left ventricle and hyperkinesis of the basal walls with a severely reduced ejection fraction. After formalization of the amputation and cardiovascular evaluation, the patient was discharged from the hospital in stable condition 10 days later.

**Conclusion:**

Emergency physicians should be aware of the possibility of stress cardiomyopathy as a cause for acute decompensation, even in isolated extremity trauma.

## INTRODUCTION

First described in the 1990s in Japan, takotsubo or stress cardiomyopathy is an uncommon cause of acute-onset heart failure, although its pathogenesis is still poorly understood.^1^ It is a syndrome of transient left ventricular systolic dysfunction seen in the absence of obstructive coronary artery disease. Although a sudden stressful or emotional event is often a precursor to the development of this condition, up to 30% of cases have no identifiable precipitating event.^2^ The name comes from a Japanese octopus trap, which resembles the balloon-like shape often taken by the left ventricle in takotsubo cardiomyopathy. The left ventricular wall motion abnormalities seen in takotsubo cardiomyopathy typically extend beyond the distribution of a single coronary artery. Hypotheses for the pathogenesis of this condition include catecholamine excess, microvascular dysfunction, and coronary artery spasm.^3^ Epidemiologically, stress cardiomyopathy affects postmenopausal women (as in this case) disproportionately.^4^

Mayo Clinic diagnostic criteria for stress cardiomyopathy are the following: 1) transient left ventricular systolic dysfunction usually extending beyond a single coronary distribution; 2) absence of obstructive coronary disease explaining the wall motion abnormalities; 3) new electrocardiogram (ECG) abnormalities or elevation in cardiac troponins; and 4) absence of pheochromocytoma or myocarditis.^5^ Mortality in the International Takotsubo Registry study was reported at 4.1%.^2^ Those that survive typically recover systolic function within one to four weeks.^6^ As the name implies, stress cardiomyopathy is typically diagnosed surrounding emotionally or physiologically stressful events. This case reminds us that stress cardiomyopathy must remain on the differential even in isolated extremity trauma.

## CASE REPORT

A 63-year-old female presented to the emergency department (ED) following an accidental subtotal amputation of her right hand with a circular saw while working on a craft project with her husband. She initially presented to an outside hospital who arranged transfer to a tertiary care center by air for plastic surgery consultation and possible re-implantation. En route she had an episode of nausea and vomiting accompanied by bradycardia to the 20s. She became briefly pulseless and underwent one round of Advanced Cardiac Life Support including chest compressions, intravenous epinephrine, and intubation. She was diverted to the closest ED for stabilization where she received one unit of packed red cells for a hemoglobin of 8 grams per deciliter (g/dL) (reference range: 11.3–15.2 g/dL). She was subsequently transported to our tertiary care center by ambulance.

**Image 1:**
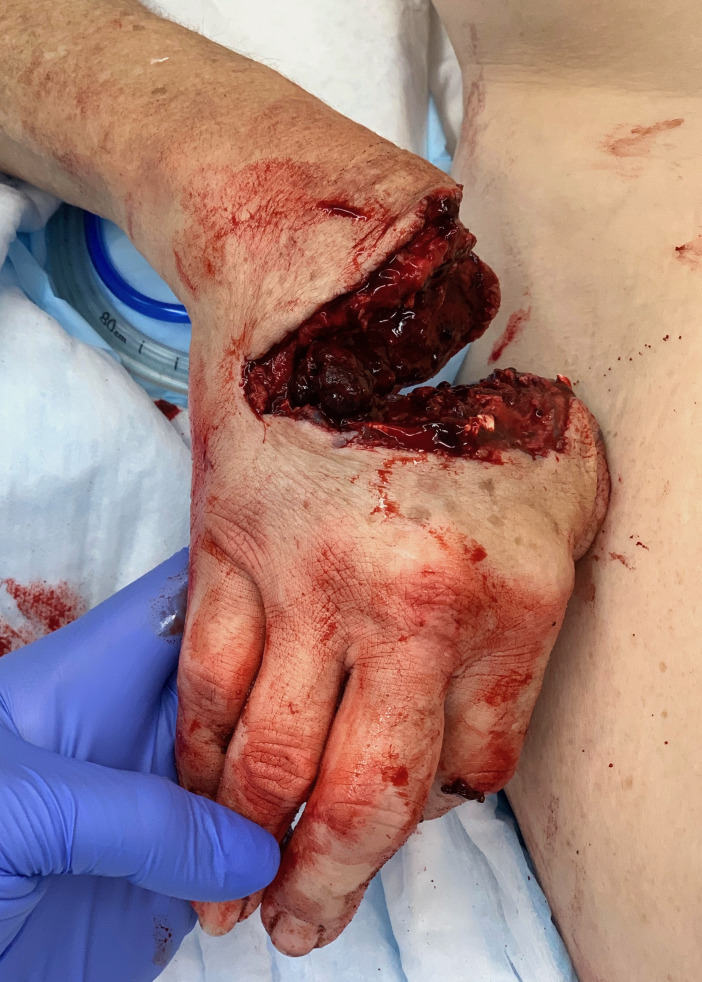
Photograph of right hand near-total amputation.

Physical examination revealed subtotal amputation through the base of all five metacarpals extending from radial to ulnar aspect with a small remaining tissue bridge along the ulnar border. An intermittent monophasic Doppler signal was present near the ulnar artery. All five fingers were cold and pale without capillary refill (Image 1).

**CPC-EM Capsule**

What do we already know about this clinical entity?

*Takotsubo or stress cardiomyopathy is typically a short-term illness, diagnosed by echocardiography. Its causes may include stress, trauma, or sudden illness*.

What makes this presentation of disease reportable?

*We describe a novel association between severe extremity trauma complicated by cardiac arrest with the rapid development of takotsubo cardiomyopathy*.

What is the major learning point?

*In the trauma or post-cardiac arrest patient, point-of-care ultrasound (POCUS) may yield findings that could have important implications for subsequent patient care*.

How might this improve emergency medicine practice?

*The use of POCUS, as well as the recognition and diagnosis of takotsubo cardiomyopathy, can expedite appropriate patient management and specialty referral*.

**Image 2:**
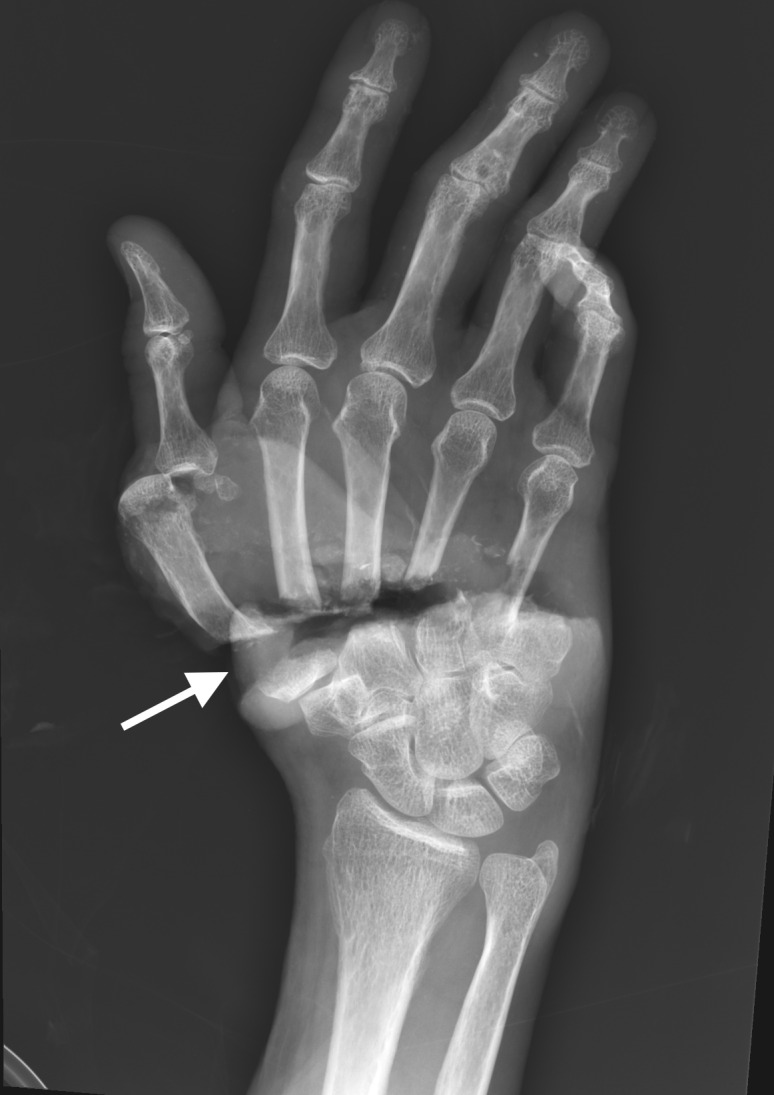
Apical 4-chamber ultrasound view demonstrating apical ballooning (arrow).

On arrival, laboratory evaluation revealed a hemoglobin of 10.7 g/dL, lactic acid of 3.7 millimoles per liter (mmol/L) (reference range: 0.5–2.2 mmol/L), and troponin of 0.183 nanograms per milliliter (ng/mL) (0.000–0.029 ng/mL). An ECG demonstrated one millimeter ST elevations in the anterior precordial leads without reciprocal depression. Point-of-care echocardiogram in the ED revealed severely reduced left ventricular ejection fraction of 25–30% with apical hypokinesis, basal hyperkinesis, mild mitral regurgitation, and preserved right heart function. This presentation and echocardiographic findings were highly concerning for takotsubo cardiomyopathy (Image 2, Video).

Following completion of the patient’s amputation at the bedside by plastic surgery, the patient was admitted to the medical intensive care unit for further management. Formal echocardiography approximately 24 hours after presentation revealed moderately reduced left ventricular systolic function with an ejection fraction of 35–40%. The mid to distal septal, anterior, and lateral walls were severely hypokinetic to akinetic with akinetic apex. The right ventricle remained normal in size and function. Troponin peaked at 1.589 ng/mL and B-type natriuretic peptide uptrended from 39 picograms per milliter (pg/mL) on arrival to 536 pg/mL (reference range: 0.0–100.0 pg/ml).

Left heart catheterization was performed on hospital day one and revealed minimal non-obstructive coronary artery disease with luminal irregularities and elevated left ventricular end diastolic pressure of 19 millimeters of mercury (mm Hg) (<12 mm Hg), essentially ruling out coronary artery disease as a cause for her cardiomyopathy. She underwent formalization of her right hand amputation with plastic surgery and was subsequently extubated. She was discharged from the hospital after a 10-day admission. Eight months following the accident the patient was fitted with a bioelectric hand prosthesis. Unfortunately, no further information was available regarding her cardiovascular recovery.

## DISCUSSION

We present the case of a previously healthy 63-year-old female who developed stress cardiomyopathy and brief cardiac arrest following isolated severe extremity trauma. Her initial hemodynamic instability and cardiovascular collapse could not be fully explained by hypovolemic shock secondary to hemorrhage. Point-of-care ultrasound (POCUS) in the ED revealed the classically described wall motion abnormalities of apical ballooning of the left ventricle and hyperkinesis of the basal walls. Diagnosis of takotsubo cardiomyopathy was later confirmed by comprehensive echocardiogram and coronary catheterization revealing an absence of significant coronary artery disease.

Although relatively uncommon – studies have placed the incidence at 1–2% in patients presenting with symptoms concerning for acute coronary syndrome – takotsubo cardiomyopathy is a diagnosis most emergency physicians will encounter in their practice, although it may not be easily recognizable.^7^,^8^ Our patient fits the classic demographic, as the syndrome disproportionately affects post-menopausal females. In the International Takotsubo Registry study, 88.9% of the affected patients were females and the mean age was 66.4 years.^2^ Takotsubo cardiomyopathy has been reported following multiple types of traumatic injury, including brain injury, subarachnoid hemorrhage, and burns, as well as motor vehicle collision.^9^–^11^

## CONCLUSION

In this case we describe a patient who suffered a cardiac arrest in the setting of isolated extremity trauma and then developed stress cardiomyopathy within hours of the initial insult. This patient had the classic findings of stress cardiomyopathy on bedside echocardiography in the ED: apical ballooning; basal hyperkinesis; and reduced ejection fraction. This case emphasizes the utility of POCUS in the critically ill or injured ED patient, providing important diagnostic information that may not be otherwise available. It reminds the emergency physician to maintain a broad differential even when the diagnosis seems to be apparent.

Because the patient’s stress cardiomyopathy was not immediately evident on ED arrival, POCUS enabled the clinical team to avoid potential subsequent mismanagement of undiagnosed severe cardiac disease. In the setting of post-traumatic cardiac arrest, a POCUS-guided diagnosis of new-onset cardiomyopathy may, as in this case, provide important diagnostic data to guide the patient’s subsequent ED management and hospital course.

## Supplementary Information

Video Point-of-care ultrasound demonstrating apical ballooning (arrow) and basal hyperkinesis (arrowheads), typical for takotsubo cardiomyopathy.
